# LIMA1 links the E3 ubiquitin ligase RNF40 to lipid metabolism

**DOI:** 10.1038/s41420-024-02072-6

**Published:** 2024-06-22

**Authors:** Zhan Liu, Kexin Fan, Aikedaimu Abudukeremu, Min Gao, Xinyue Tan, Xiaojuan Mao, Xinyu Li, Wenting Ma, Xusheng Ma, Caolong Li, Yinglai Yang, Kangsheng Tu, Jing Chen, Yilei Zhang, Yaqun Guan

**Affiliations:** 1 State Key Laboratory of Pathogenesis, Prevention and Treatment of High Incidence Diseases in Central Asia, School of Basic Medical Sciences, Xinjiang Medical University, Urumqi, Xinjiang China; 2 Department of Biochemistry and Molecular Biology, School of Basic Medical Sciences, Xinjiang Second Medical College, Karamay, Xinjiang China; 3 The Institute of Molecular and Translational Medicine, Department of Biochemistry and Molecular Biology, School of Basic Medical Sciences, Xi’an Jiaotong University, Xi’an, Shaanxi China; 4 Department of Hepatobiliary Surgery, the First Affiliated Hospital of Xi’an Jiaotong University, Xi’an, Shaanxi China; 5 Department of Obstetrics, Xi ’an New Chang ’an Maternity Hospital, Xi’an, Shaanxi China

**Keywords:** Mechanisms of disease, Ubiquitylation

## Abstract

LIMA1 is a LIM domain and Actin binding 1 protein that acts as a skeleton protein to promote cholesterol absorption, which makes it an ideal target for interfering with lipid metabolism. However, the detailed regulation of LIMA1 remains unclear. Here, we identified that ring finger protein 40 (RNF40), an E3 ubiquitin ligase previously known as an epigenetic modifier to increase H2B ubiquitination, mediated the ubiquitination of LIMA1 and thereby promoted its degradation in a proteasome-dependent manner. Fraction studies revealed that the 1–166aa fragment of LIMA1 was indispensable for the interaction with RNF40, and at least two domains of RNF40 might mediate the association of RNF40 with LIMA1. Notably, treatment with simvastatin dramatically decreased the levels of CHO and TG in control cells rather than cells with overexpressed LIMA1. Moreover, RNF40 significantly decreased lipid content, which could be reversed by LIMA1 overexpression. These findings suggest that E3 ubiquitin ligase RNF40 could directly target LIMA1 and promote its protein degradation in cytoplasm, leading to the suppression of lipid accumulation mediated by LIMA1. Collectively, this study unveils that RNF40 is a novel E3 ubiquitin ligase of LIMA1, which underpins its high therapeutic value to combat dysregulation of lipid metabolism.

## Introduction

Cholesterol is an essential structural component of cell membranes and a synthetic precursor of steroid hormones as well as vitamin D, which maintains the metabolic processes of many biological functions [1, 2]. Deregulated cholesterol metabolism has been linked to several prevalent diseases, such as diabetes mellitus, atherosclerosis, and neurodegenerative disease [3]. As the major tissue for cholesterol absorption, intestine accounts for roughly 50% of the daily cholesterol input within our bodies [4]. Several key proteins mediate the cholesterol absorption and transport in specific organs. Niemann-Pick C1 Like 1 (NPC1L1), expressed in liver and intestine, is the key transmembrane protein known to promote cholesterol uptake via a vesicular transport mechanism [5], which make it a therapeutic target for the clinical manipulation of cholesterol level. Indeed, the drug ezetimibe inhibits NPC1L1 leading to a significant reduction in cholesterol absorption, ultimately decreasing blood cholesterol level [6]. In fact, the function of NPC1L1 is tightly regulated by its associated proteins. LIM domain and Actin binding 1 (LIMA1), which interacts with NPC1L1 and regulates its trafficking by recruiting myosin Vb to promote cholesterol absorption [7]. Notably, a rare frameshift variant in the LIMA1 identified from a Chinese family of Kazakh ethnicity causes low cholesterol and reduced cholesterol absorption in the carriers, which is also observed in LIMA1-deficient mice [7]. These findings demonstrate that LIMA1 is a key protein regulating cholesterol absorption, highlighting an additional target for manipulating cholesterol level besides NPC1L1.

LIMA1, also known as EPLIN or SREBP3, is initially identified as an actin-binding protein that is selectively produced in human epithelial cells [7–9]. Previous studies revealed that LIMA1 negatively correlates with invasiveness, metastasis, poor prognosis, mortality and therapeutic resistance in human tumors such as prostate, breast, ovarian and esophageal cancers [10]. Mechanistically, epidermal growth factor (EGF) could cause protein phosphorylation and turnover of LIMA1 through an extracellular signal-regulated kinase (ERK) signal pathway to influence epithelial-mesenchymal transition (EMT) [11]. Besides, Rab40b–Cullin5-dependent ubiquitylation regulates LIMA1 localization by altering focal adhesion and cytoskeletal dynamics [12]. These studies suggest that LIMA1 might play a critical role in the regulation of cytoskeleton and relevant cellular functions such as cholesterol metabolism and structural remodeling. However, the detailed mechanisms underlying the regulation of LIMA1 protein remain elusive. A better understanding of the molecular regulation of LIMA1 protein level including protein stability modulation, could help explore more therapeutic means to target LIMA1-mediated physiological functions such as cholesterol metabolism. Notably, the Leu to Ile mutation of the 25th amino acid in LIMA1 protein (L25I) is also revealed in Chinese Kazakhs, who obviously have a lower plasma cholesterol concentration. Further studies found that L25I mutation partially impairs the cholesterol absorption function of LIMA1 possibly through inhibiting the protein stability of LIMA1, though the underlying mechanism remain unclear [7]. It indicates that LIMA1 protein stability might be tightly regulated in vivo and disruption of its stability could participate in the regulation of cholesterol metabolism.

Proteosome-mediated protein degradation is a major way to control protein stability. Ring finger family (RNF) proteins act as E3 ligase to promote ubiquitin-chain elongation-mediated degradation of target proteins, including the critical factors involved in the lipid metabolism. For example, ring finger protein 5 (RNF5) activates sterol regulatory element-binding protein 2 (SREBP2) to promote cholesterol biosynthesis via inducing polyubiquitination of SREBP chaperone SCAP [13]. Ring finger protein 145 (RNF145) is recognized by a ubiquitin ligase for sterol-induced degradation of HMG-CoA reductase [14]. Previous study suggests that LIMA1 protein stability is tightly regulated, since LIMA1–L25I mutant rather than its wild-type is prone to be degraded [7]. Thus, whether proteosome-mediated protein degradation participates in the regulation of LIMA1 stability remain further investigation.

In this study, we set out to explore the E3 ubiquitin ligase that modulates LIMA1 stability by pulling-down Flag-tagged LIMA1 protein followed by mass spectrometry analysis. Among the LIMA1-associated proteins, RNF40 was identified as a key E3 ligase to decrease LIMA1 protein stability through proteosome-mediated degradation. The current knowledge of RNF40 focus on its role in H2B monoubiquitylation, DNA damage repair, apoptosis, and cancer development [15]. Here, we reported that RNF40 accelerates the degradation of LIMA1 and regulates cell sensitivity to lipid synthesis inhibitor simvastatin through modulating LIMA1 protein level.

## Results

### LIMA1 stability is regulated by ubiquitin-mediated proteasome degradation

Previous study indicates that LIMA1 protein is highly expressed in the tissues of liver and intestine [7]. Thus, we detected the expression of LIMA1 in different cell lines originated from liver, and high expression of LIMA1 was observed in LO2, HepG2, Bel-7402, PLC and Huh6 cells (Fig. S1A). To investigate whether LIMA1 is regulated by the proteasome pathway, we treated LO2, Bel-7402 and SMMC-7721 cell lines with proteasome inhibitor MG132 for 12 h, and found that LIMA1 protein level was upregulated, which is similar to the change of HIF2α that is known to be degraded by proteosome and severs as a positive control upon MG132 treatment (Fig. S1B). Subsequently, we treated Bel-7042 cells together with the MG132 and cycloheximide (CHX), an inhibitor of protein synthesis, to determine the protein stability of LIMA1. The result showed that LIMA1 protein levels were generally higher upon MG132 treatment compared to that of vehicle-treated cells, while HIF2α level is more stable when treated with MG132 as expected (Fig. S1C).

**Fig. 1:**
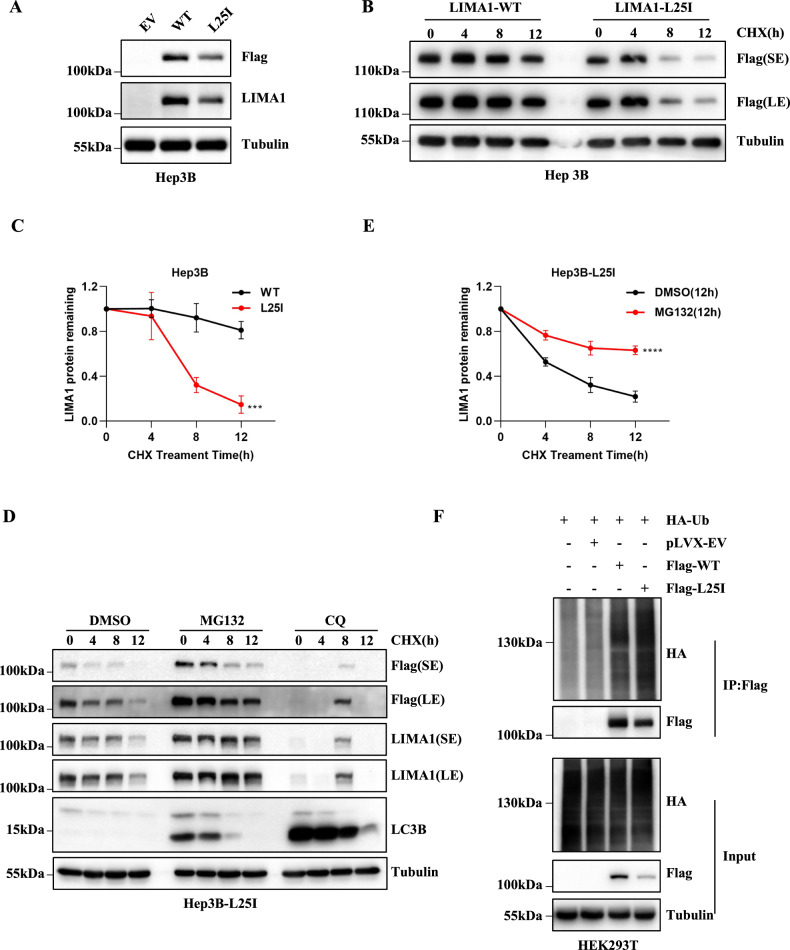
LIMA1 stability is regulated by ubiquitin-mediated proteasome degradation. **A** The expression of exogenous LIMA1-WT and LIMA1-L25I mutant in Hep3B cells was detected by immunoblotting. **B**, **C** Hep3B cells overexpressed with LIMA1 or L25I were treated with 50 μM CHX, and samples were collected at the indicated time points for Western blotting with indicated antibodies (**B**). Relative levels of LIMA1 at different time points were plotted (**C**). *n* = 3 independent repeats per group. *P* value was calculated using two-way ANOVA. ***p < 0.001. **D**, **E** Hep3B-L25I cells were treated with CHX for the indicated time in the presence of dimethyl sulfoxide (DMSO), 10 μM MG132 or 50 μM chloroquine (CQ) followed by Western blotting analysis (**D**). Relative levels of LIMA1 at different time points were plotted. *n* = 3 independent repeats per group (**E**). *P* value was calculated using two-way ANOVA. ****p < 0.0001. **F** HEK293T cells were transfected with indidated plasmids followed by MG132 treatment for 8 h before collecting samples, then LIMA1 protein was immunoprecipitated with anti-Flag antibody for further immunoblotting assay.

To further study the regulatory mechanism of protein degradation for exogenous LIMA1, we constructed stable cell lines overexpressed with wild-type LIMA1 (WT) or LIMA1-L25I mutant (L25I) in cell lines with low expression of LIMA1, including SMM7721, SNU449, BEL-7402, and Hep3B, while the expression of WT and L25I seems no difference at the basal level (Fig. S1D and Fig. 1A). Then, we examined the protein stability of the exogenous LIMA1 protein in the presence of CHX, and found that L25I is less stable than WT (Fig. 1B, C), which is consistent with previous findings [7]. Next, we employed the proteasome inhibitor MG132 or autophagy inhibitors chloroquine (CQ) to determine the regulatory pathway decreasing the stability of L25I. The results clearly showed that MG132 rather than CQ dramatically promoted the stability of LIMA1-L25I protein, while LC3B protein level is higher in the presence of CQ, suggesting that proteosome- rather than autophagy-mediated degradation is responsible for the downregulation of LIMA1 stability (Fig. 1D, E). More importantly, the immunoprecipitation results indicated that MG132 treatment increased the total ubiquitination levels of WT and L25I in HEK293T cells (Fig. 1F). Interestingly, the ubiquitin level of L25I was obviously higher than that of WT, regardless of the fact that the total level of L25I was lower than that of WT (Fig. 1F). These findings indicate that LIMA1 degradation is controlled by the ubiquitination-mediated proteasome pathway, but the exact enzyme regulating its ubiquitin modification is unclear.

### Identification of RNF40 as an E3 ligase to promote proteosome-mediated degradation of LIMA1

**Fig. 2:**
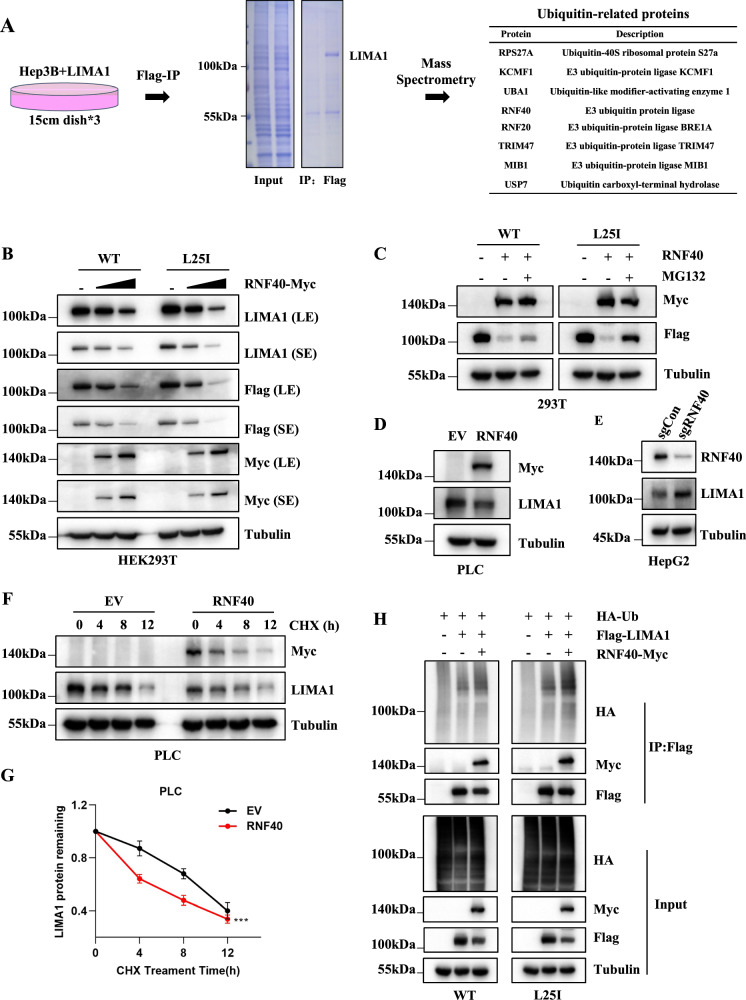
Identification of RNF40 as an E3 ligase to promote proteosome-mediated degradation of LIMA1. **A** Mass spectrometry (MS) identification of LIMA1-associated ubiquitin-related proteins. Schematic work flow and SDS-PAGE gel verification of immunoprecipitated proteins. **B** HEK293T cells were transfected with Flag-LIMA1 or L25I and different amounts of RNF40-Myc followed by immunoblotting analysis. **C** LIMA1-WT or LIMA1-L25I mutant were transfected into HEK293T cells with or without RNF40 for 36 h, then samples were collected after MG132 treatment for 8 h and analyzed by Western blotting. **D**, **E** Western blotting analysis of RNF40-overexpressed PLC cells (**D**) and RNF40-konckout HepG2 cells (**E**). **F**, **G** LIMA1 protein stability was determined in PLC cells overexpressed with RNF40 in the presence of CHX (**F**). Relative levels of LIMA1 at different time points for the two cell lines were plotted (**G**). *n* = 3 per group. *P* values was calculated using two-way ANOVA. ***p < 0.001. **H** HEK293T cells were transfected with HA-Ub, LIMA1-WT/L25I, and RNF40 followed by MG132 treatment for 8 h, then the samples were collected for anti-Flag immunoprecipitation and immunoblotting analysis.

To search for the key enzymes that modulate the ubiquitin level of LIMA1, we performed immunoprecipitation using Anti-Flag antibody in Hep3B cells overexpressed with Flag-LIMA1 (WT) or Flag-LIMA1-L25I (L25I) to capture the interacting partners of LIMA1 protein. The immunoprecipitated protein complexes were then resolved in SDS-PAGE followed by mass spectrometric analysis to identify each associated protein (Fig. 2A). Interestingly, there were 8 proteins involved in the regulation of protein ubiquitination level including E3 ligase like RNF20, RNF40, KCMF1, TRIM47, MIB1, and deubiquitinating enzyme USP7 (Fig. 2A). Next, we transfect exogenous RNF20 or RNF40 into HEK293T cells and examine the expression levels of wild-type (WT) or mutant (L25I) LIMA1 protein. Overexpression of RNF20 rarely had effect on LIMA1 expression (Fig. S2A). Of note, the expression level of either LIMA1-WT or LIMA1-L25I was obviously down-regulated by RNF40 in a concentration-dependent manner (Fig. 2B). Interestingly, the level of LIMA1-L25I protein seemed to decrease faster compared with that of LIMA1-WT (Fig. S2B). Then, we determined the level of LIMA1 regulated by RNF40 in the presence of MG132, and found that both WT and L25I mutant expression levels could be largely restored after adding MG132 (Figs. 2C and S2C), suggesting that RNF40 suppresses LIMA1 expression via proteosome-mediated protein degradation. Then we generated stable cell line with RNF40-overexpression in PLC cells and found endogenous LIMA1 protein was significantly downregulated (Figs. 2D and S2D). Conversely, knockout RNF40 through CRISPR technology in HepG2 cells upregulated LIMA1 protein expression (Figs. 2E and S2E). To access the protein stability of LIMA1 in the presence of RNF40, we treated the cells with CHX, a protein synthesis inhibitor, and observe the protein level of LIMA1 at indicated time points. The data showed that RNF40 significantly decreased the expression level of LIMA1 compared to control cell line without exogenous RNF40 expression (Fig. 2F, G). More importantly, to test whether RNF40 mediates the ubiquitination of LIMA1, immunoprecipitation of LIMA1 in HEK293T cells overexpressed with RNF40 and ubiquitin was performed followed by immunoblot analysis, and it showed that RNF40 significantly increased the polyubiquitination level of LIMA1-WT as well as LIMA1-L25I (Fig. 2H). Altogether, these data strongly suggested that RNF40 regulates the stability of LIMA1 through ubiquitination–proteasome mediated degradation.

### N-terminal region of LIMA1 is required for its association with RNF40

**Fig. 3:**
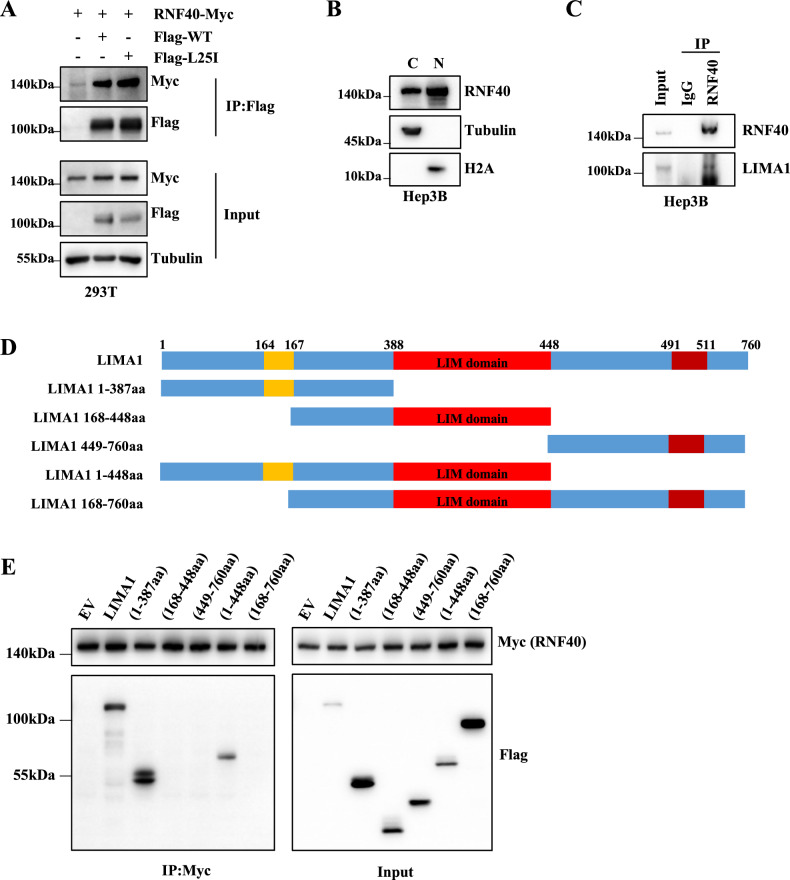
N-terminal region of LIMA1 is required for its association with RNF40. **A** Flag-LIMA1 or L25I and RNF40-Myc were co-expressed in HEK293T cells followed by co-immunoprecipitation with Anti-Flag antibody and immunoblotting analysis. **B** Nuclear and cytoplasmic protein extraction was performed and the separated proteins were subjected for immunoblotting analysis. **C** Co-IP was performed for RNF40 in Hep3B cells and LIMA1 protein was detected by immunoblotting. **D** Schematic diagram of full-length LIMA1 and their various deletion mutants. **E** HEK293T cells were co-transfected with RNF40-Myc and different LIMA1 mutants, followed by immunoprecipitation with anti-Myc antibody and immunoblotting analysis.

To verify the findings that RNF40 acts as an E3 ligase to regulated LIMA1 protein stability through protein-protein interaction, we next studied the association pattern between RNF40 and LIMA1-WT or LIMA1-L25I. As shown in Fig. 3A, immunoprecipitation of Flag-tagged LIMA1-WT or LIMA1-L25I could also pull-down Myc-tagged RNF40, indicating that either wild-type or mutant LIMA1 could interact with RNF40. However, RNF20, another protein also identified from MS analysis, could interact with LIMA1-WT or LIMA1-L25I as well (Fig. S3A), though LIMA1 protein level was not affected (Fig. S2A). LIMA1 is known as a cytoskeleton protein, while RNF40 has been shown to locate in both cytoplasm and nucleus [16]. We did cellular fraction analysis and indeed found the cytoplasmic and nuclear localization of endogenous RNF40 (Fig. 3B). Next, a specific antibody against RNF40 was employed to pull-down endogenous RNF40, and endogenous LIMA1 expression was clearly detected (Fig. 3C), suggesting the interaction between LIMA1 and RNF40 within liver cells. Then, different Flag-tagged truncates of LIMA1 were generated (Fig. 2D) and transfected into HEK293T cells together with RNF40-Myc. Western blotting analysis of immunoprecipitated proteins revealed that only 1–387aa and 1–448aa of LIMA1 could interact with RNF40, while 168–448aa, 449–760aa, and 168–760aa of LIMA1 lost the ability to interact with RNF40. These results imply that 1–166aa fragment of LIMA1 are indispensable for the interaction with RNF40 (Fig. 3E). Similarly, we generated different RNF40-Myc truncates (Fig. S3C) and transfected them together with Flag-LIMA1 into HEK293T cells. The Co-immunoprecipitation data indicated that all the RNF40 truncations still interacted with LIMA1 (Fig. S3D), which suggests that at least two domains of RNF40 could mediate the association of RNF40 with LIMA1.

### RNF40 regulates lipid metabolism by suppressing LIMA1

**Fig. 4:**
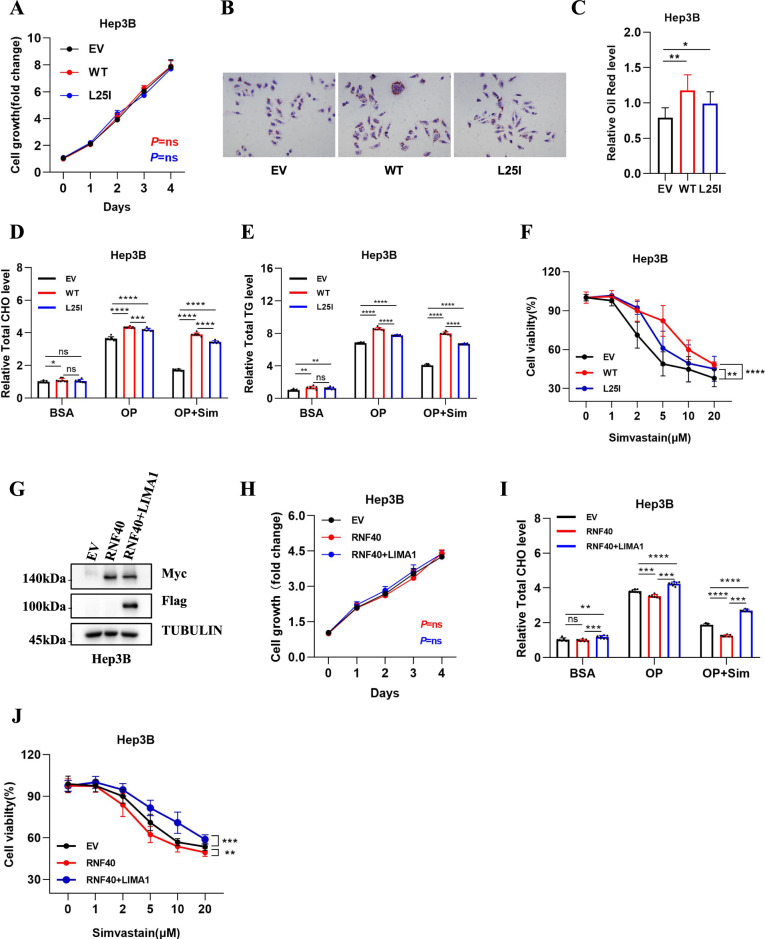
RNF40 regulates lipid metabolism through suppressing LIMA1. **A** Cell growth of indicated cell lines. *n* = 4 independent repeats. ns, no significant. **B**, **C** Oleic acid (0.66 μM) and palmitic acid (0.33 μM) were added to induce lipogenesis in Hep3B cells with LIMA1-WT or L25I mutant for 24 h, then the cells were subsequently stained with oil red. The intensity and areas of staining was analyzed by ImageJ software. *n* = 10 random photos captured for each group. Error bars are mean ± sd., *p < 0.05; **p < 0.01. **D**, **E** The contents of total cholesterol (CHO) or triglyceride (TG) in indicated Hep3B cell lines under different treatment conditions were determined. BSA, 5% bovine serum albumin; OP, 0.66 μM oleic acid and 0.33 μM palmitic acid; OP + Sim, 0.66 μM oleic acid, 0.33 μM palmitic acid and 10 μM simvastatin. *n* = 6 independent repeats per group. Error bars are mean ± sd., *p < 0.05; **p < 0.01; ***p < 0.001; ****p < 0.0001. **F** Curve graphs showing the viability of the indicated cells treated with various concentrations of simvastatin for 48 h. n = 4 independent repeats. *P* values calculated using Two way ANOVA. n = 4 per group. **p < 0.01; ****p < 0.0001. **G** Verification of Hep3B cell lines with overexpression of RNF40 and LIMA1. **H** Cell growth analysis of cell lines generated in Fig. 4G. **I** The total cholesterol content was determined in the indicated Hep3B cell lines with various treatments. BSA, 5% bovine serum albumin; OP, 0.66 μM oleic acid and 0.33 μM palmitic acid; OP + Sim, 0.66 μM oleic acid, 0.33 μM palmitic acid and 10 μM simvastatin. *n* = 6 independent repeats. Error bars are mean ± sd., **p < 0.01; ***p < 0.001; ****p < 0.0001. **J** Cell viability of the indicated cell lines treated with the different concentrations of simvastatin for 48 h. *n* = 4 independent repeats. *P* values calculated using two way ANOVA. **p < 0.01; ***p < 0.001.

To study the cellular function regulated by LIMA1, we firstly examined the cell proliferation ability in the indicated cell lines and found that neither LIMA1-WT nor L25I mutant had any effect on cell growth (Fig. 4A). Previous findings demonstrated that LIMA1 interacts with NPC1L1 and facilitates cholesterol uptake [17]. Thus, we detected lipid content in these cells using oil red staining assay and found that LIMA1 could significantly increase lipid content, while the upregulation of lipid content by LIMA1-L25I is not as dramatic as LIMA1-WT (Fig. 4B, C). Subsequently, we examined total cholesterol in different cell lines and found that cholesterol (CHO) levels were significantly higher in LIMA1-WT cells than in the other two groups (Fig. 4D), and similar changes were observed for triglyceride (TG) levels (Fig. 4E). Moreover, treatment with simvastatin dramatically decreased the levels of CHO and TG in EV cells rather than cells with overexpression of LIMA1-WT or -L25I (Fig. 4D, E). This observation prompts us to explore the detrimental effects of simvastatin on these cell lines since simvastatin is known to suppress cell growth and induce cell death [18]. Indeed, we treated the cells with simvastatin at indicated concentration for 48 h, and found that cell viability was dramatically decreased, while LIMA1-WT seemed to cause stronger resistance to simvastatin treatment compared with LIMA1-L25I (Fig. 4F). To determine whether RNF40 could regulated lipid metabolism through LIMA1, we established stable cell line with expression of both RNF40 and LIMA1 (Fig. 4G). Overexpression with RNF40 alone or together with LIMA1 had no effect on cell proliferation (Fig. 4H). However, RNF40 could significantly decrease CHO level, which was restored by LIMA1 overexpression, suggesting that RNF40 downregulated CHO level through the suppression of LIMA1 (Fig. 4I). Besides, RNF40 combined with simvastatin treatment dramatically decreased CHO level, which was also revered by restored LIMA1 expression (Fig. 4I). Consistently, RNF40 also sensitized cells to simvastatin treatment-induced cell death, which was largely ameliorated by LIMA1 restoration (Fig. 4J). The results demonstrated that LIMA1 promotes lipid synthesis and resistance to simvastatin treatment, which is negatively regulated by RNF40 through the suppression of LIMA1.

### Multi-omics analysis revealed increased de novo synthesis of triacylglycerol and glycerophosphate esters upon overexpression of LIMA1

**Fig. 5:**
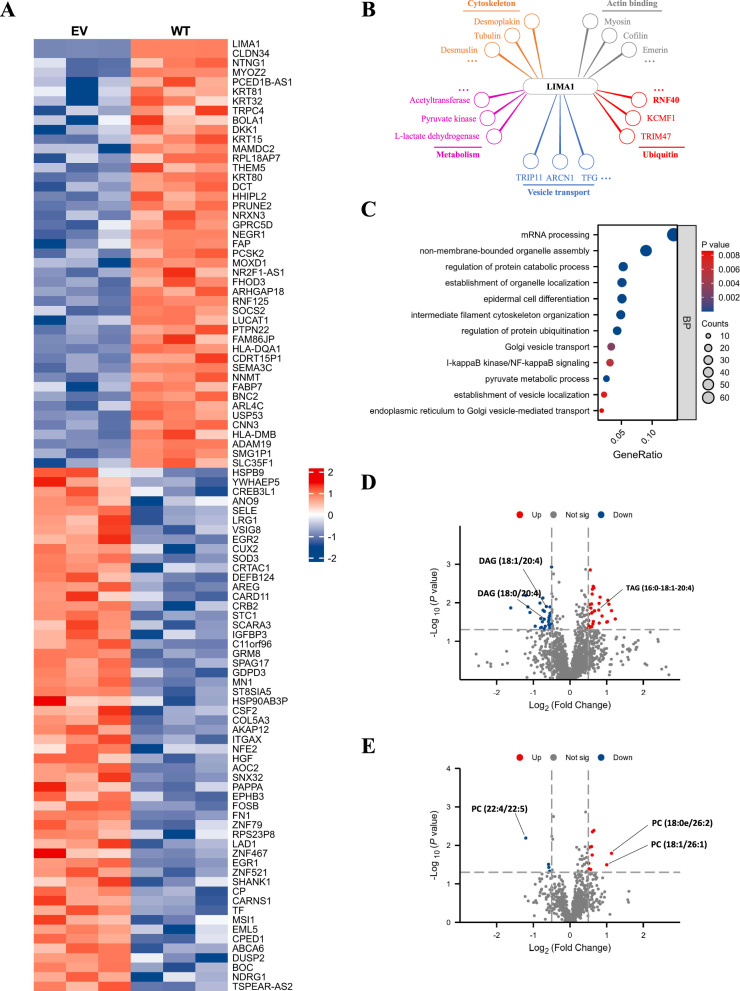
Multi-omics analysis revealed increased de novo synthesis of triacylglycerol and glycerophosphate esters upon overexpression of LIMA1. **A** Heatmap showing the RNA-seq analysis results of the top 100 dysregulated genes upon LIMA1 overexpression in Hep3B cells. **B** Groups of putative LIMA1-associated partners identified by mass spectrometry analysis. **C** Gene ontology (GO) enrichment analysis of interactome for LIMA1. **D**, **E** Lipidomic analysis of LIMA1-overexpressed Hep3B cells compared with control cells. The dysregulated glycerides (**D**) and phospholipids (**E**) were highlighted in separated volcano plot.

To explore the underlying mechanism for the regulation of lipid metabolism by LIMA1, we performed RNA-seq analysis for control (EV) and LIMA1-overexpressed (WT) cells, and identified 261 differential expressed genes (139 upregulated genes and 122 downregulated genes) potentially regulated by LIMA1 overexpression (Fig. 5A and supplementary data set 1), implying that LIMA1 has few effects on the regulation of gene transcription. Since the above findings indicated significant regulation of LIMA1 on lipid synthesis (Fig. 4B–E), we checked the expression levels of genes related to lipid metabolism and found that LIMA1 rarely affect their mRNA expression (Fig. S4A). Besides, GO analysis of the RNA-seq results showed that the upregulated molecules were mainly involved in epidermis development, intermediate filament-based process and cell adhesion (Fig. S4B). These results indicate that LIMA1 might modulate lipid-related metabolism independent of transcriptional regulation. The aforementioned results have explored LIMA1-associated proteins using Anti-Flag magnetic beads-mediated immunoprecipitation followed by mass spectrometry (Fig. 2A), which revealed five main groups of potential interaction partners with LIMA1 [19]: cytoskeletal proteins (e.g., tubulin and desmoplakin), actin-binding factors (e.g., myosin and cofilin), vesicle transport (e.g., TFG and ARCN1), enzymes involved in metabolism (e.g., pyruvate kinase and acetyltransferase) and E3 ubiquitin ligases (RNF40, TRIM47 and KCMF1) (Fig. 5B and supplementary data set 2). Consistently, many of the potentially interacting molecules identified by our mass spectrometry were also identified in previous publications [7, 19]. GO analysis the interactome of LIMA1 also found enrichment of proteins in ubiquitination and vesicle transport (Fig. 5C and S4C, D). Because lipid synthesis and transport is closely controlled by actin cytoskeleton and vesical-mediated processing [13], our results suggested that LIMA1 may promote lipid metabolism through interacted proteins involved in vesicle transport, which needs further investigation.

Next, lipidomic analyzes were employed to study cellular lipids on a large scale in Hep3B-EV/WT cells. By comparing the abundance of individual lipid species, we identified 61 differential molecules among 2036 lipid chemical species (Fig. S4E), which were simply classified as glycerides, glycerophospholipids and sphingolipids. It’s noted that TAG level is significantly higher than that of the control group (Fig. 5D and supplementary data set 3), which is similar to our above findings (Fig. 4E). Moreover, the large accumulation of TAG correspondingly led to a decrease in the level of DAG in Hep3B cell (Fig. 5D), which is in line with that TAG is mainly synthesized from DAG in hepatocytes and adipose tissue through the diglyceride pathway [20]. In addition, serving as the most important glycerophosphate esters in cell membrane components, 10 different species were identified by MS and 9 of them were significantly increased especially for PC (18:1/26:1) and PC (18:0e/26:2) (Fig. 5E). Though lipidomic characterization did not find any significant difference in sterol lipid profiles (Fig. S4F), LIMA1 dramatically upregulated the levels of sphingolipid components, including SM (d30:2/13:0) and SM (d25:1/19:0) (Fig. S4G). In general, these data suggest that LIMA1 might regulate the triglyceride and glycerophosphate esters accumulation through interacted proteins related to cytoskeleton and vesicular transport, while the detailed molecular mechanisms remain further investigation.

## Discussion

LIMA1 has been widely recognized for its role in regulating lipid metabolism [7, 17, 21, 22], but how LIMA1 is tightly modulated at the post-translational level remains unclear. In this study, we aimed to decipher the detailed mechanisms underlying the regulation of LIMA1 stability. First, we demonstrate that LIMA1 protein stability is controlled by proteasome-mediated protein degradation (Fig. 1). Through unbiased analysis of IP-MS assay, we identified ubiquitination system-related proteins and confirmed that RNF40 acts as an E3 ubiquitinase to decrease LIMA1 stability in a manner of ubiquitin-mediated protein degradation. Moreover, we explored lipid content and found LIMA1 significantly promoted lipid accumulation, which could be the reason that LIMA1-overexpressed cells were more resistant to simvastatin-induced suppression of liver cells. Though LIMA1 rarely affected gene expression, interactome and lipidomic analyzes of cells with or without LIMA1 overexpression suggested that LIMA1 could promote the accumulation of triglyceride and glycerophosphate esters, while the detailed mechanisms behind the lipid metabolism regulation by LIMA1 remain further investigation.

During anabolic processes lipids, the most important players are the synthetic rate-limiting enzymes HMG-CoA and squalene monooxygenase [4]. SREBP2, serving as an ER-anchored precursor, associated with SCAP to form a protein complex and moves from the ER to the Golgi for proteolytic activation upon lipids depletion. The activated SREBP2 designated nuclear SREBP2 (nSREBP2), then enters the nucleus as a homodimer, binds to the sterol regulatory element (SRE) sequence in the promoters of target genes, including *HMGCR* and *SQLE* (encoding squalene monooxygenase), and upregulates their transcription to upregulate lipid synthesis [23]. However, based on our analysis of transcriptional genomics, LIMA1 (also known as SERBP3) couldn’t modulate the mRNA levels of lipid biosynthesis-related enzymes, suggesting alternative ways of lipid metabolism regulation by LIMA1.

In recent years, ubiquitination has emerged as an important mode of post-translational regulation of cellular cholesterol homeostasis. Zhang reported that RNF145 induces polyubiquitination of SCAP and thus inhibits cholesterol biosynthesis [24]. Ring finger protein 5 (RNF5) mediates the Lys-29-linked polyubiquitination of SCAP and thereby activates SREBP2 [13]. INSIGs are absolutely required for the sterol-induced degradation of HMGCR [25]. When cellular cholesterol is deficient, GP78 and TRC8 target ubiquitination INSIG1 to promote its degradation, thereby enabling SCAP-SREBP2 transport from ER to the Golgi with COPII vesicles [26, 27]. In turn, when cellular cholesterol is abundant, INSIG1 binds to SCAP-SREBP2, making it unable to follow COPII vesicles to Golgi, at the same time, the two key enzymes of cholesterol synthesis, HMG-CoA and squalene monooxygenase, were degraded by E3 ligase GP78, TRC8, RNF145, or MARCH6, which inhibited the synthesis of cholesterol [14, 28]. However, the ubiquitination modulating enzymes targeting LIMA1 is totally unknow. Here, we proved that RNF40 might acts as an E3 ubiquitinating enzyme targeting LIMA1 for polyubiquitination-mediated protein degradation (Fig. 2B-H). Interestingly, mutant LIMA1 (L25I) appeared to be more unstable compared to its wild-type, which is not due to the capacity of its association with RNF40 (Fig. 3A). It is still challenging to elucidate the reason why LIMA1-L25I is more easily to be degraded.

**Fig. 6:**
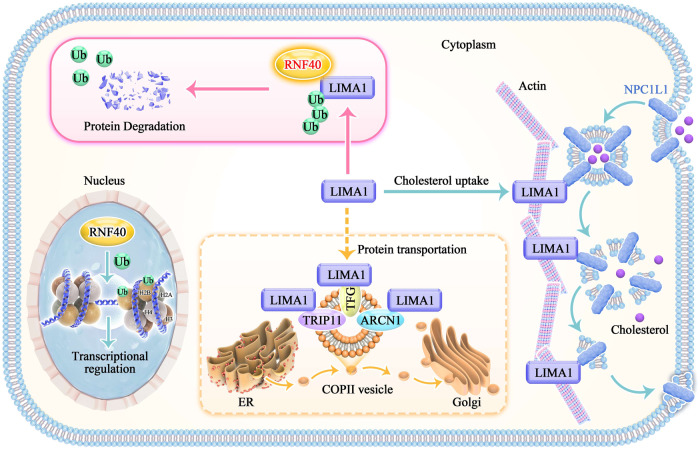
LIMA1 stability is regulated by ubiquitin-mediated proteasome degradation. Proposed working model for LIMA1 and lipid metabolism regulation by RNF40.

RNF40 has been demonstrated to monoubiquitinate histone H2B at lysine 120 (H2Bub1) via its E3 ligase activity, which is required for gene regulation at the epigenetic level and essential for several biological functions, including cell reprogramming and metabolism [29–31]. Particularly, loss or low expression of RNF40 is negatively correlated with the expression levels of genes enriched in glycolysis, which supports a pivotal role of RNF40 in maintaining the glycolytic program and promoting the malignant behavior of breast cancer [31]. However, less is known about the role of RNF40 in the cytoplasm and its potential targets involved in the regulation of lipid metabolism. Indeed, we found obvious cytoplasmic localization of RNF40, which could interact with LIMA1 and promote its degradation in a proteasome-dependent manner (Figs. 2 and 3). Moreover, RNF40 regulates lipid accumulation and increases the sensitivity of cells to simvastatin-induced growth inhibition through targeting LIMA1 (Fig. 4). These findings possibly reveal that RNF40 could engage its E3 ligase activity to promote protein degradation independent of H2Bub1 and gene transcription regulation (Fig. 6), which indicates a previously uncovered role of RNF40 in the regulation of gene expression.

## Materials and methods

### Cell lines

The liver cancer cell lines and the HEK293T embryonic kidney cell line were gifts from Zhang lab at The Institute of Molecular and Translational Medicine, Xi’an Jiaotong University. All cell lines were cultured in DMEM containing penicillin (100 units/mL), streptomycin (100 mg/mL), and 10% (vol/vol) FBS except for LO2, which were cultured in RPMI-1640 medium. Cells within eight passages were used for experiments. All cell lines were tested annually to be Mycoplasma-free Stable cell lines were generated as previously described [32]. Briefly, HEK293T cells were transfected with individual plasmids targeting specific genes together with the psPAX.2 and pMD2.G third-generation lentiviral packaging systems by using Lipofectamine 3000 reagent according to the manufacturer’s instructions. At 36–48 h later, lentivirus particles in the medium were collected and filtered before being used to infect cell lines in the presence of 8 μg/mL Polybrene. At 24 h after infection, puromycin was added at a concentration of 2 μg/mL to obtain stable cell lines with successful transduction. To generate CRISPR/Cas9-mediated knockout cells, the single-guide RNA (sgRNA) targeting the specific gene was cloned into the lentiCRISPR v2 (Addgene, #52961). Oligonucleotide and primer sequences were as follows: Control sgRNA forward, 5-CACCGGCACTACCAGAGCTAACTCA-3; Control sgRNA reverse, 5-AAACTGAGTTAGCTCTGGTAGTGCC-3; RNF40 sgRNA1 forward, 5-CACCGCCAGGCAACAAACGCGCCGC-3; RNF40 sgRNA1 reverse, 5-AAACGCGGCGCGTTTGTTGCCTGGC-3. To confirm knockout efficiency, expression levels of target genes were determined by immunoblotting with corresponding antibodies.

### Reagents

MG132 is a potent proteasome inhibitor (Med Chem Express, HY-13259), Cycloheximide is an antifungal antibiotic (Sigma-Aldrich, 239763-M), Oleic Acid and palmitic acid (Sigma-Aldrich, O1008, W283207), Lipofectamine 3000 reagent (Thermo Fisher Scientific, L3000015), Puromycin (Thermo Fisher Scientific, A1113803), Polybrene (Beyotime Biotechnology, C0351), Normal rat IgG (Beyotime Biotechnology, A7031), Protein A/G Magnetic Beads (Beyotime Biotechnology, P2108), Anti-Flag and Anti-Myc Magnetic Beads (Beyotime Biotechnology, P2115, P2118).

### Oil red O staining

Prepare 1.5H (170 ± 20 µm) cover glasses were placed into a 6-well plate and the medium was removed when the cell growth confluence reached 2/3. Submerse the sample in freshly prepared 3.7% formaldehyde solution for 10 min at room temperature and wash twice in deionized water, blot off excess fluid but do not let the sample dry out completely. Immediately stained with oil red O solution (Beyotime Biotechnology, C0158S) for 10 min at room temperature. Rinse cells in 85% propylene glycol solution for 30 seconds and wash twice in deionized water. Submerse the sample in freshly hematoxylin solution for 10 min at room temperature. Images were acquired with Olympus BX51 microscopy after thoroughly rinsing the samples with tap water. Finally, images data were quantified using ImageJ v1.46r software.

### Western blot analysis

Protein levels were determined by immunoblotting as previously described [33]. Briefly, Cultured cells were lysed with Nonidet P-40 buffer (150 mM sodium chloride, 1.0% Nonidet P-40, and 50 mM Tris) containing complete mini protease inhibitors (Roche) and phosphatase inhibitor mixture (Calbiochem). Western blots were obtained utilizing 20-40 ng of lysate protein. The following antibodies were used in this study: anti-Beta Tubulin antibody (Proteintech, 66240, 1:10,000 dilution), LIMA1 rabbit mAb (Thermo Fisher Scientific, PA5-31567, 1:1000 dilution), RNF40 antibody rabbit mAb (ABclonal Technology, A6443, 1:1000 dilution), anti-Flag and anti-Myc tag rabbit mAb (Cell Signaling Technology, 14793 S, 2278 S, 1:1000 dilution), anti-HA tag mouse mAb (OriGene, TA100012, 1:1000 dilution).

### Co-immunoprecipitation and mass spectrometry

Hep3B cell lines were stable expressing LIMA1/l25I-Flag. The expression of exogenous protein LIMA1/L25I was confirmed by immunoblotting. For affinity purification, a total of three 15 cm dishes of Hep3B cells expressing Flag-tagged LIMA1 were lysed in NP40 buffer containing protease inhibitors for 20 min at 4 °C. Crude lysates were cleared by centrifugation, and the supernatants were incubated with 100 μl anti-Flag magnetic beads 8 h at 4 °C. The beads were washed three times with NP40 buffer, the bound proteins were eluted by boiling in SDS sample buffer, resolved by SDS-PAGE, visualized by Coomassie blue staining and subjected to mass spectrometric analysis (Applied Protein Technology Mass Spectrometry Facility at Shanghai).

### Cell viability

Cell viability was measured as described previously [34]. Briefly, 5000 cells per well were seeded in 96-well plate and treated, after which, the medium in each well was replaced with fresh medium containing Cell Counting Kit-8 reagent (Top science, C0005). After incubation for 1 h at 37 °C, plates were read by a microplate reader (TECAN infinite M200Pro) at an absorbance of A450 nm. To measure cell proliferation, 1500 cells per well were seeded in a 96-well plate. At 24 h after seeding, cell viability was measured using CCK8 reagent as described above. This was considered day 0. Later, cell viability was analyzed every 24 h, and absorbance was normalized to that measured on day 0. Cell growth was indicated by the fold change from day 0 to as long as day 4, and was graphed.

### Total cholesterol/TG measurement

Triglyceride assay kit and Total cholesterol assay kit (Nanjing Jiancheng, A110-2-1, A111-2-1) was used to measure the triglyceride and total cholesterol of cells. Two hundred thousand cells were seeded into 6-well plate overnight. After removing the medium, OA + PA medium was used to induce lipogenesis for 24 h. Removing the medium, the cells were washed three times with PBS for collected, and then the operation was completed according to the instructions.

### RNA sequencing and bioinformatic analysis

RNA was isolated from stably expressed line cells using the Total RNA Extraction Reagent following the manufacturer’s protocol (ABclonal, RK30129). Samples were collected in duplicate independently. RNA-Seq was performed at the Beijing Tsingke Biotech Co., Ltd. The raw reads in FASTQ format were aligned to the reference genome, hg19, using the MOSAIK-implemented Smith-Waterman algorithm to perform a gapped alignment. The overlaps between aligned read and annotated genes, gene code V15, were counted using HTSeq software. The reads mapped to both strands were counted. The gene counts were normalized using the scaling factor method. Under any condition, if the number of overlapped reads of any given gene is less than one per million total mapped reads for all samples, this gene was excluded from further analysis. Cluster Profiler R package was used to performed Gene Ontology (GO) enrichment analysis of DEGs, in which gene length bias was corrected. GO terms with corrected *P* value less than 0.05 were considered significantly enriched by DEGs. Cluster Profiler R package was used to test the statistical enrichment of DEGs in KEGG pathways.

### Quantification and statistical analysis

Results of cell culture experiments were obtained from at least 3 independents repeats. For all statistical analyses, the difference was considered significant with a *P* value < 0.05. Comparisons between two conditions or groups were analyzed by two-tailed Student’s t tests in GraphPad Prism 8 (GraphPad Software, Inc.). Two-way analysis of variance was used to calculate differences between two curves with multiple time or concentration points. Data are presented as mean ± S.D., with at least three biologically independent replicates in each group. The detailed statistic for each plot was described in figure legends.

### Supplementary information

Supplementary figure and data sets legends

Supplementary Figure 1

Supplementary Figure 2

Supplementary Figure 3

Supplementary Figure 4

Supplementary data set 1

Supplementary data set 2

Supplementary data set 3

Supplementary uncropped blots

## Data Availability

All data are available upon reasonable request. Please contact the corresponding author for any data inquiries.
