# Supplementary material for: LIMA1 links the E3 ubiquitin ligase RNF40 to lipid metabolism
Source: Cell Death Discov. 2024 Jun 22;10:298. doi: 10.1038/s41420-024-02072-6 (PMC11193757; doi:10.1038/s41420-024-02072-6)
Supplement: Supplementary file 1 — Supplementary figure and data sets legends [file 41420_2024_2072_MOESM1_ESM.doc]

**Fig. S1 LIMA1 stability is regulated by ubiquitin-mediated proteasome degradation**

**A** Expression of LIMA1 in different hepatocellular carcinoma cell lines.

**B** Expression of LIMA1 in the indicated cell lines with or without 10 μM MG132.

**C** Hep3B, BEL-7402, SUN449 or SMMC7721 cells were treated with 50 μM CHX for the indicated time in the presence of dimethyl sulfoxide (DMSO) or 10 μM MG132. Then the protein expression level was determined by immunoblotting.

**D** Generation of LIMA1-overexpressed cell lines with wild-type (WT) or L25I mutant expression in SMMC7721, SUN449 and Huh7.

**Fig. S2 Identification of RNF40 as an E3 ligase to promote proteosome-mediated degradation of LIMA1**

**A, B** HEK293T cells were transfected with Flag-LIMA1/L25I and different amounts of RNF20-Myc, and analyzed by Western blotting (A). The ImageJ software was used to quantify LIMA1 protein abundance, and the relative level of LIMA1 protein was plotted (B). n = 3 independent repeats. *P* value was calculated using two-way ANOVA. ****p* < 0.001.

**C** Quantification analysis of the protein abundance of LIMA1 WT or L25I upon RNF40 overexpression with or without 10 μM MG132 treatment. n = 3 independent repeats per group. Error bars are mean ± sd., **p* < 0.05; ***p* < 0.01; ****p* < 0.001.

**D, E** The relative level of LIMA1 protein in the generated cell lines as indicated. n = 3 independent repeats per group. Error bars are mean ± sd., **p* < 0.05; ***p* < 0.01.

**Fig. S3 N-terminal region of LIMA1 is required for its association with RNF40**

**A** The interactions between RNF20-Myc and Flag-LIMA1/L25I were observed by Co-IP in HEK293T.

**B** The schematic diagram of different fragments of RNF40.

**C** The interactions between full-length LIMA1 and truncated forms of RNF40-Myc were analyzed by Co-immunoprecipitation in HEK293T cells.

**Fig. S4** **Multi-omics analysis revealed increased de novo synthesis of triacylglycerol and glycerophosphate esters upon overexpression of LIMA1**

**A** The mRNA levels of lipid synthesis related genes in LIMA1-overexpressed Hep3B and control cells from RNA-seq data analysis.

**B** GO analysis for the 139 upregulated genes of Hep3B cells upon LIMA1 overexpression.

**C D** “Molecular function” (C) and “Cell component” (D) from GO analysis for LIMA1-associated proteins identified from mass spectrometry.

**E** Heatmaps showing the profile of 61 dysregulated lipid molecules from lipidomic analysis in Hep3B cells upon LIMA1 overexpression.

**F G** Lipidomic analysis showing changes of CE (F) and SM (G) in Hep3B cells upon LIMA1 overexpression.

**Supplementary data set 1**

Deregulated genes after RNA-seq analysis of Hep3B cells with LIMA1-overexpression (WT) versus control (EV) cells.

**Supplementary data set 2**

LIMA1-associated proteins explored by anti-flag magnetic beads-mediated immunoprecipitation followed by mass spectrometry analysis.

**Supplementary data set 3**

Lipidomic analysis of deregulated lipid species in Hep3B-WT/EV cells.
