# Supplementary figures and images for: LIMA1 links the E3 ubiquitin ligase RNF40 to lipid metabolism
Source: Cell Death Discov. 2024 Jun 22;10:298. doi: 10.1038/s41420-024-02072-6 (PMC11193757; doi:10.1038/s41420-024-02072-6)

A

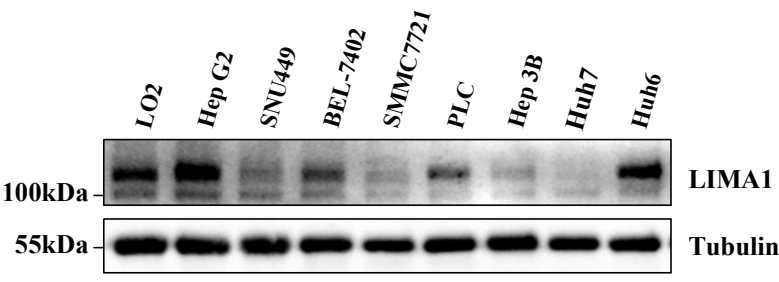

B

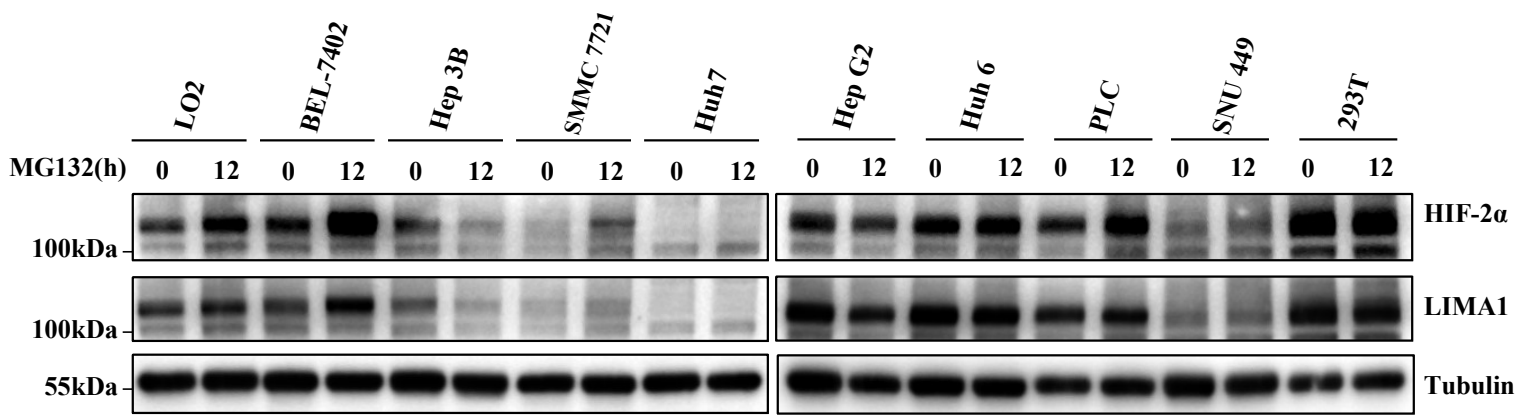

C

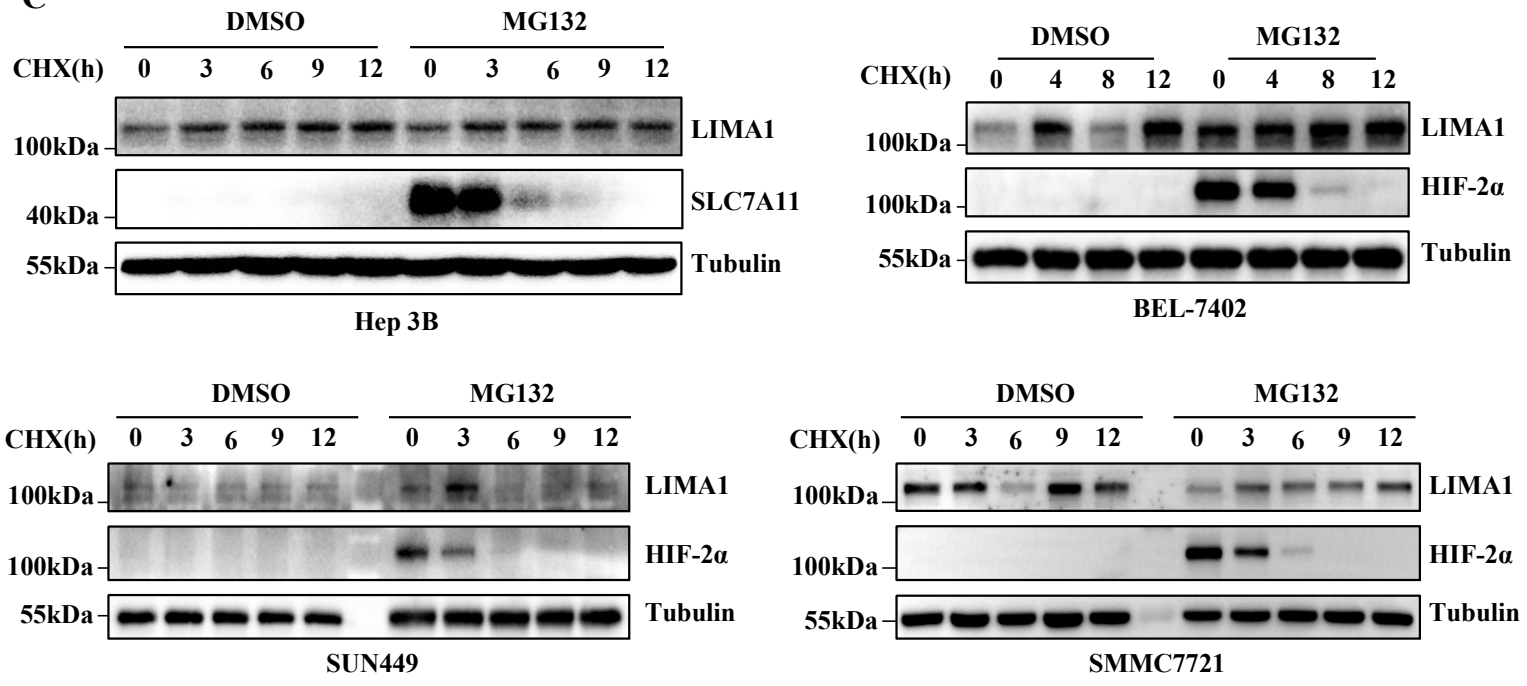

D

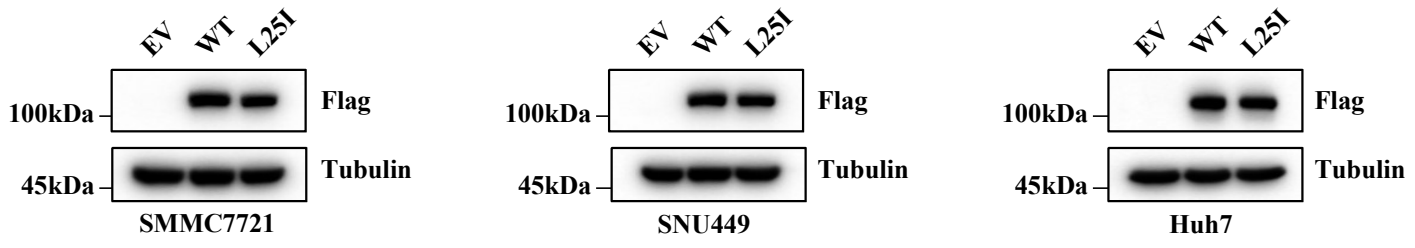

Supplement: Supplementary file 2 — Supplementary Figure 1 [file 41420_2024_2072_MOESM2_ESM.pdf]

**A**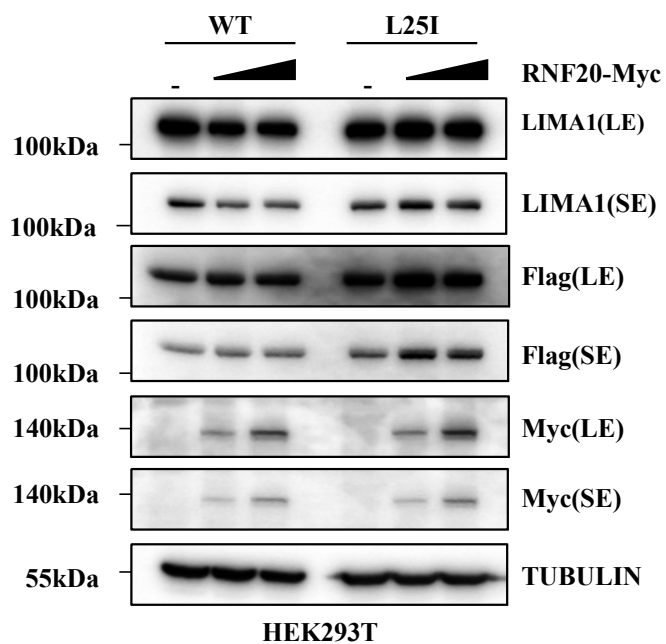**B**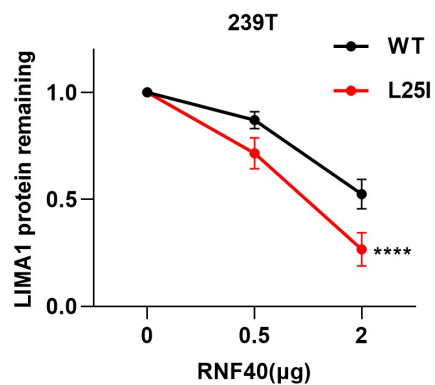**C**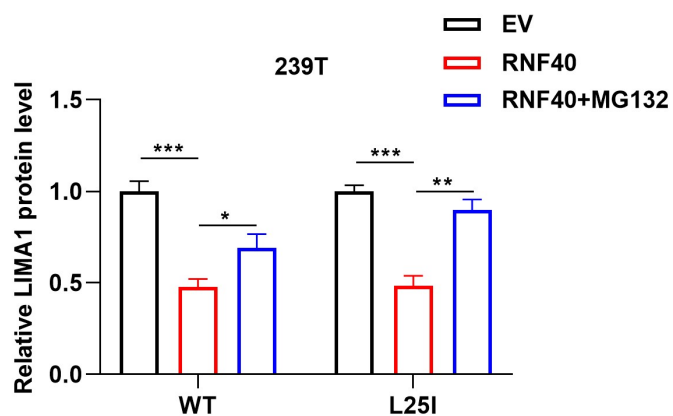**D**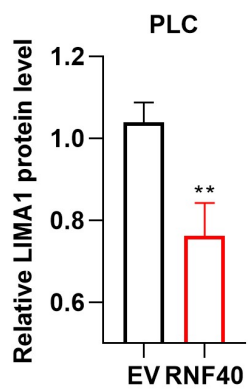**E**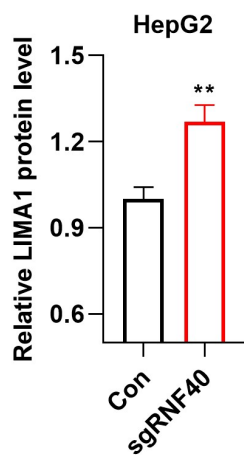

Supplement: Supplementary file 3 — Supplementary Figure 2 [file 41420_2024_2072_MOESM3_ESM.pdf]

**A**

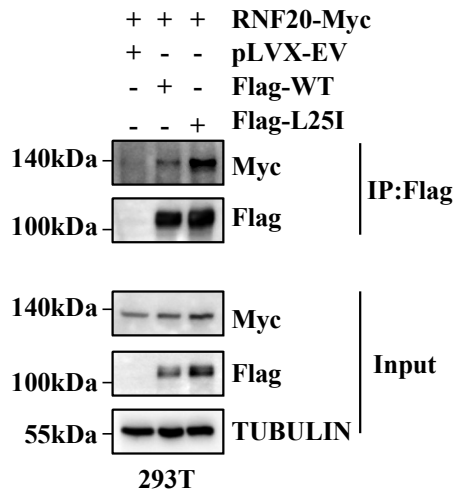

**B**

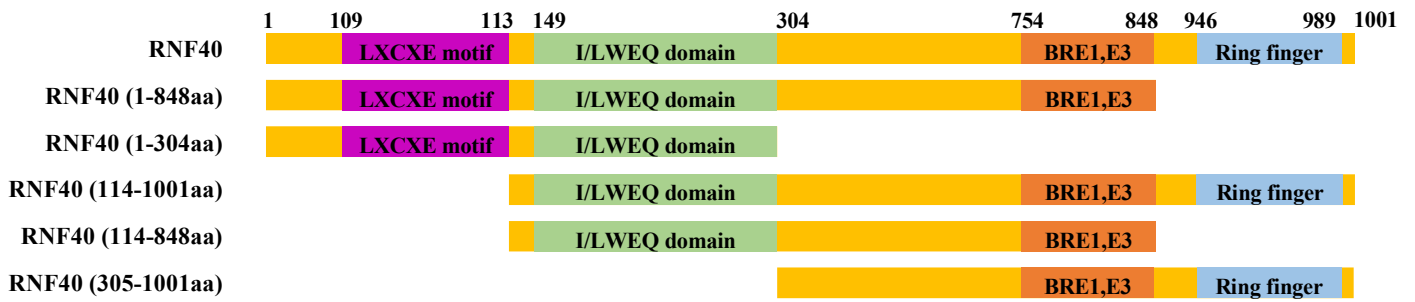

**C**

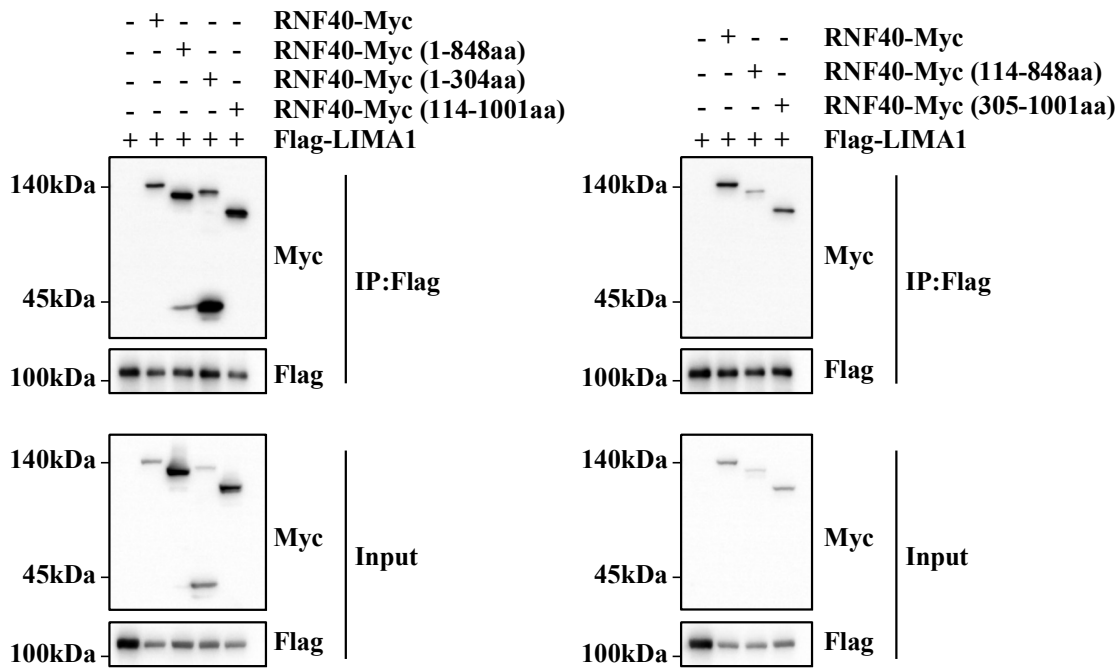

Supplement: Supplementary file 4 — Supplementary Figure 3 [file 41420_2024_2072_MOESM4_ESM.pdf]

**A**

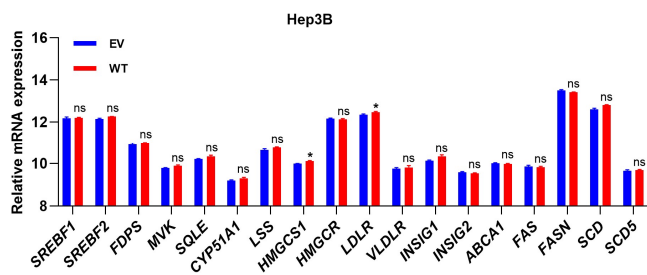

**B**

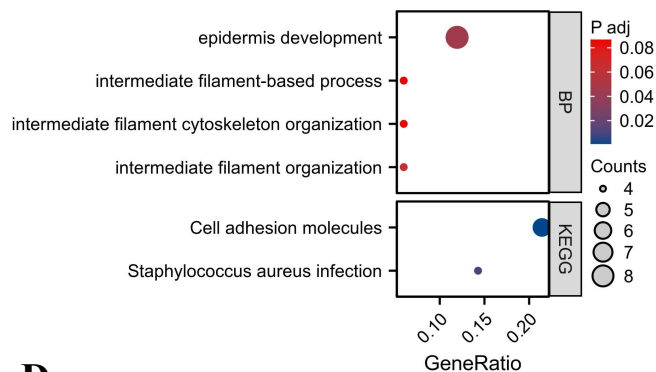

**C**

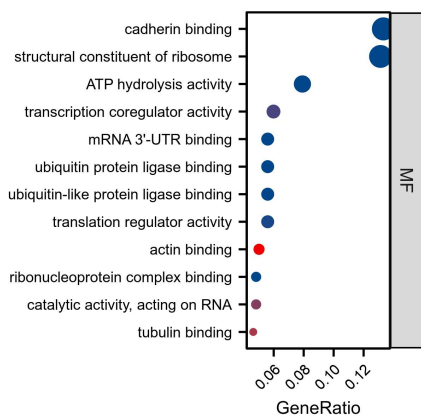

**D**

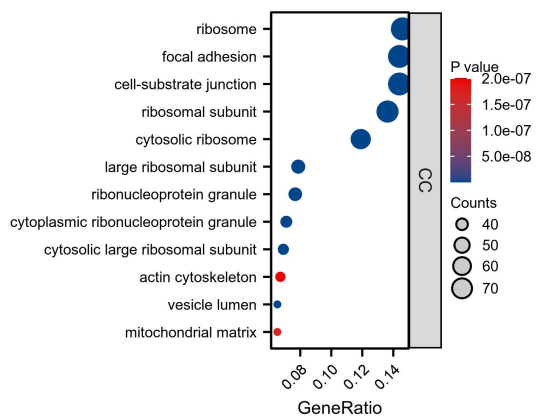

**E**

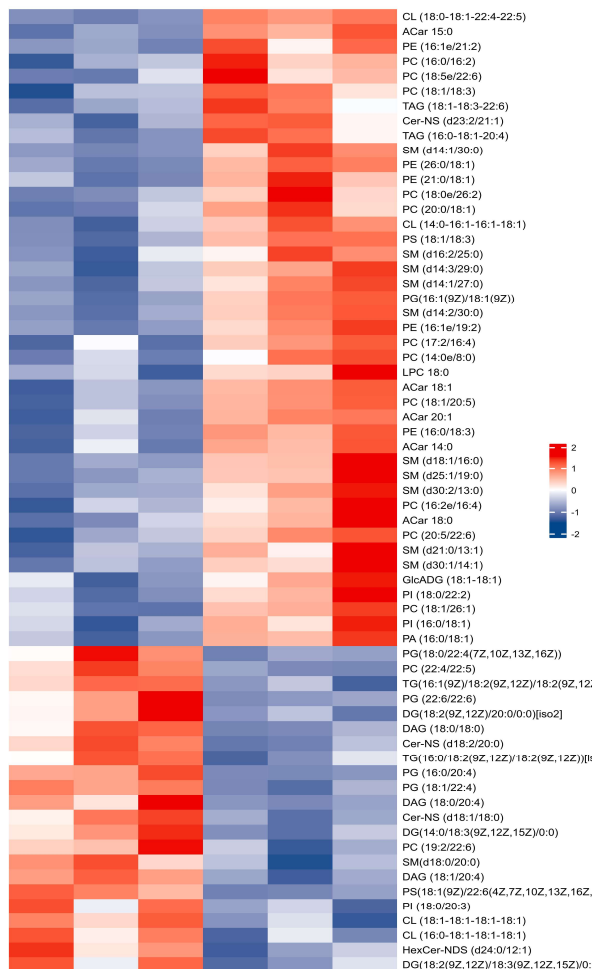

**F**

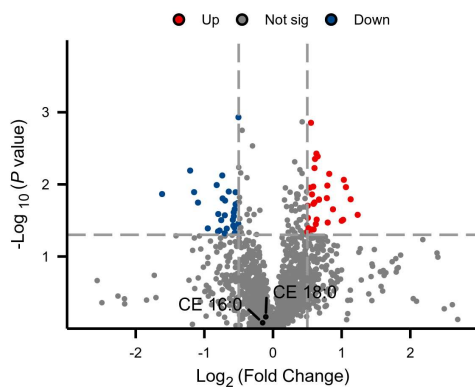

**G**

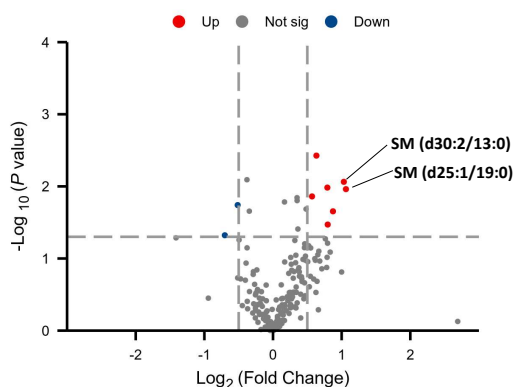

Supplement: Supplementary file 5 — Supplementary Figure 4 [file 41420_2024_2072_MOESM5_ESM.pdf]
