# Supplementary material for: LIMA1 links the E3 ubiquitin ligase RNF40 to lipid metabolism
Source: Cell Death Discov. 2024 Jun 22;10:298. doi: 10.1038/s41420-024-02072-6 (PMC11193757; doi:10.1038/s41420-024-02072-6)

Fig.1 A

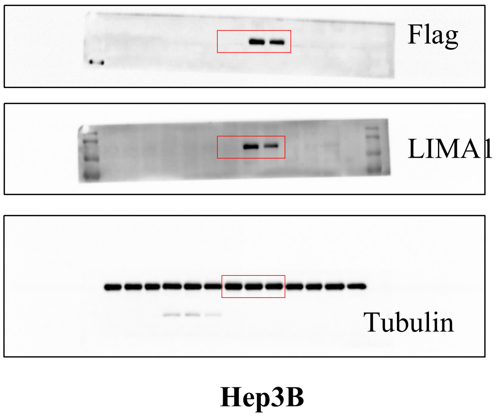

Fig.1 B

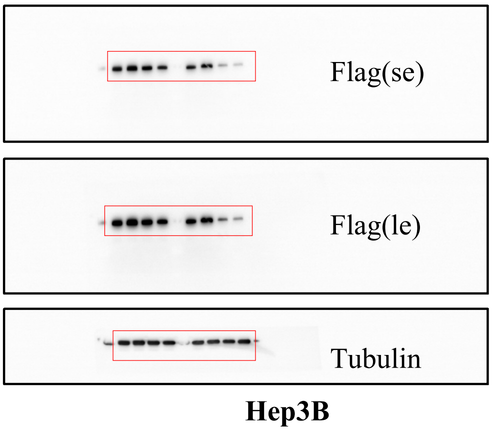

Fig.1 D

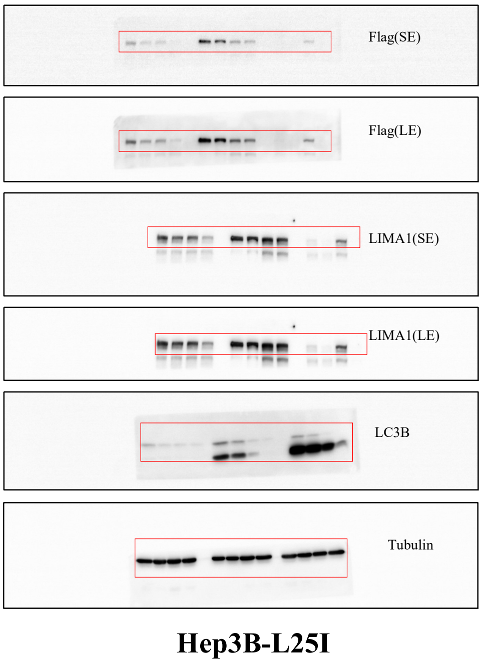

Fig.1 F

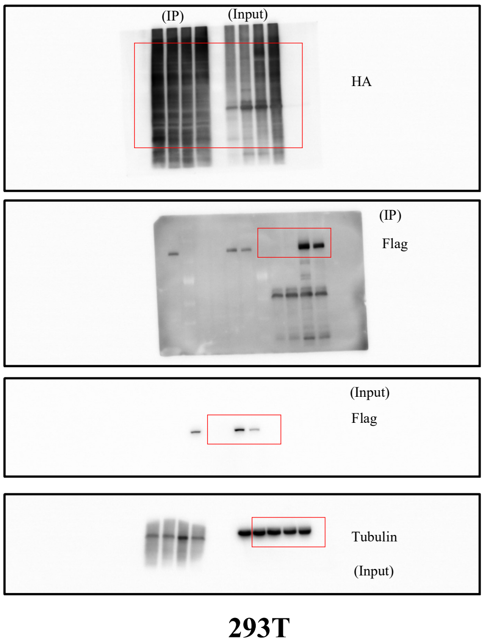

Fig.2 A

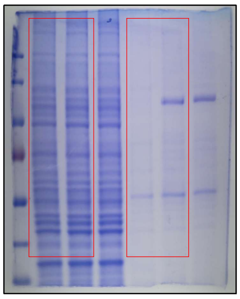

Hep3B

Fig.2 C

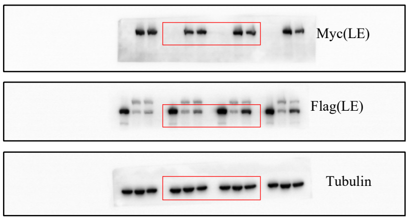

293T

Fig.2 D

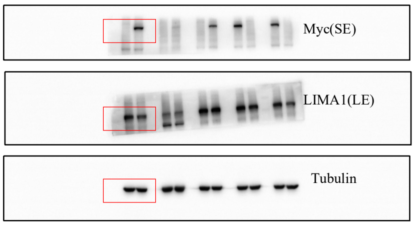

PLC

Fig.2 B

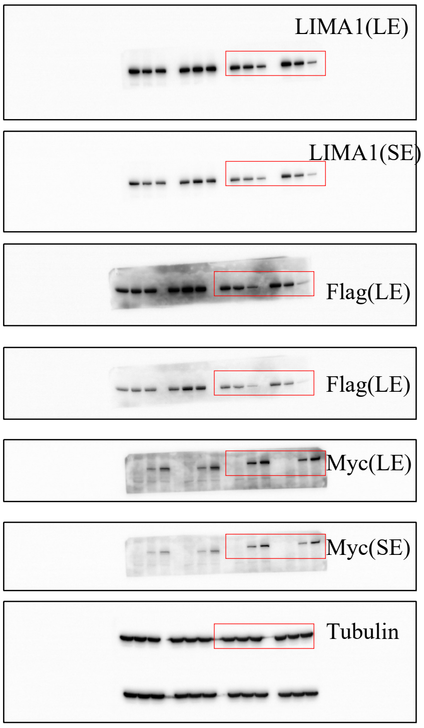

293T

Fig.2 E

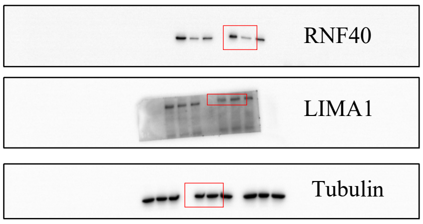

HepG2

Fig.2 H

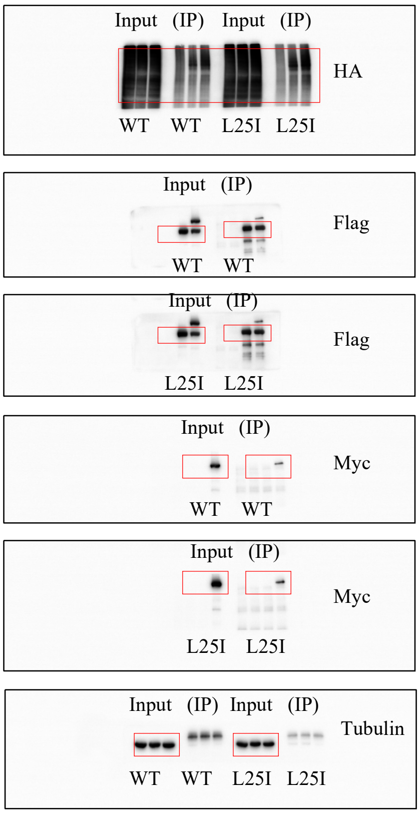

293T

Fig.2 F

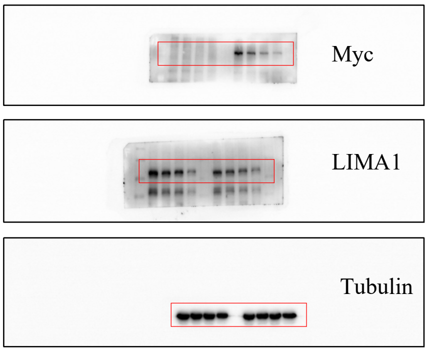

PLC

Fig.3 A

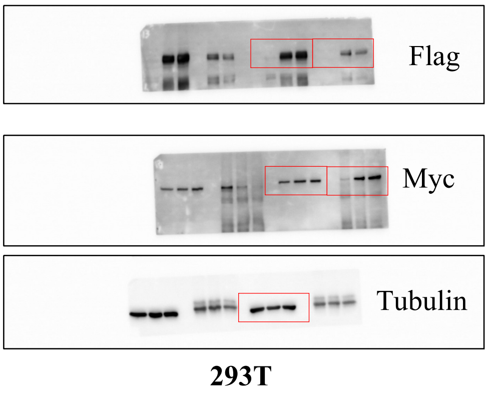

Fig.3 B

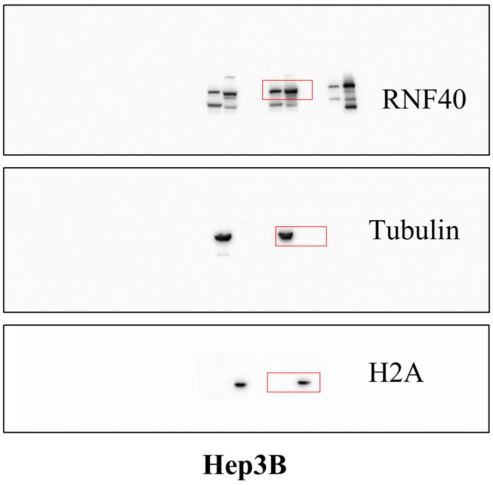

Fig.3 C

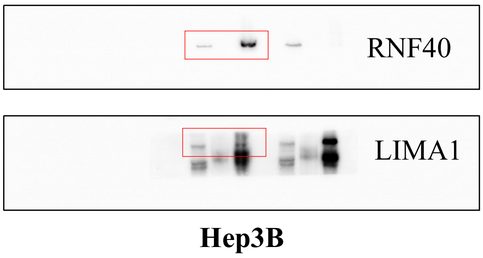

Fig.3E

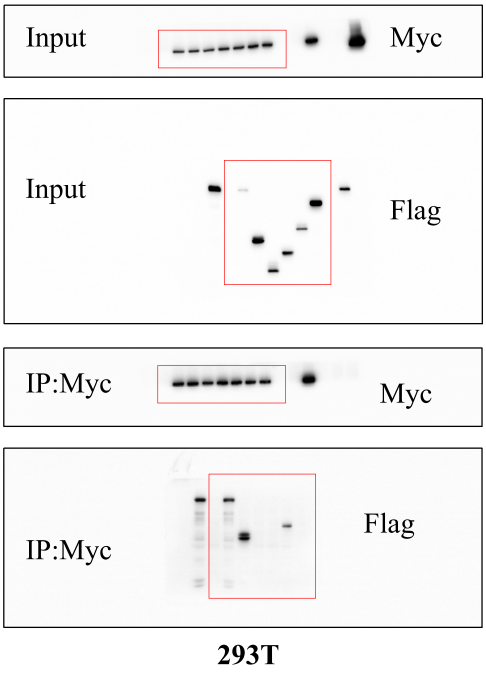

Fig.4 G

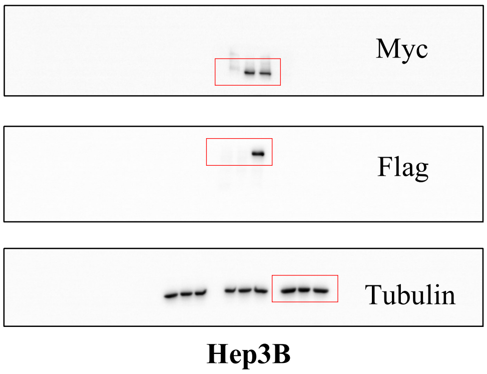

Fig.S1 A

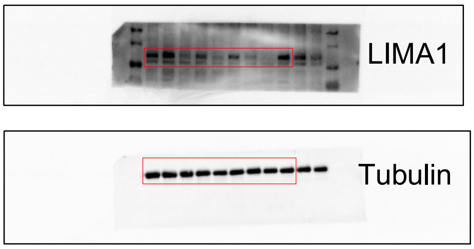

Fig.S1 B

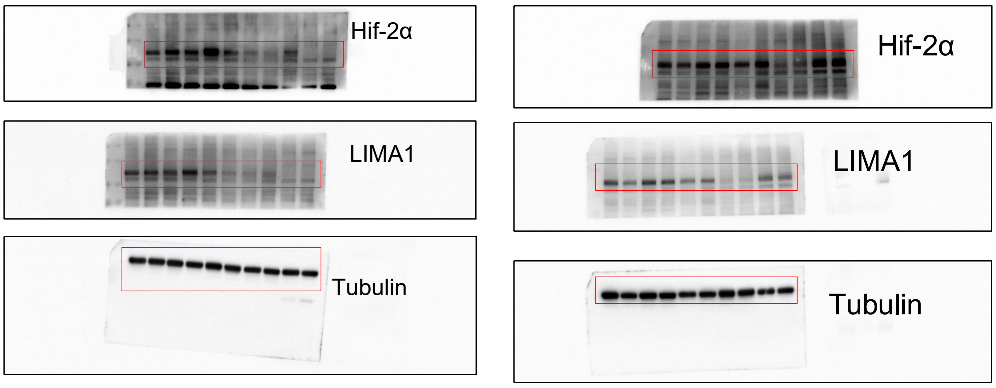

Fig.S1 C

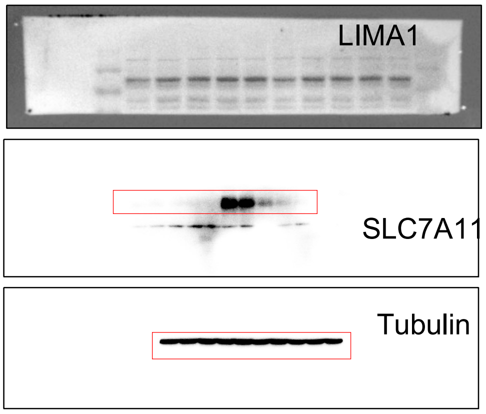

Hep3B

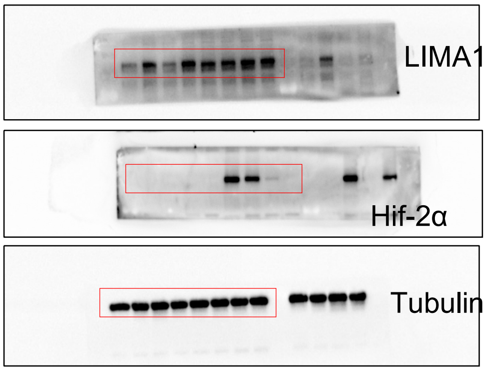

BEL-7402

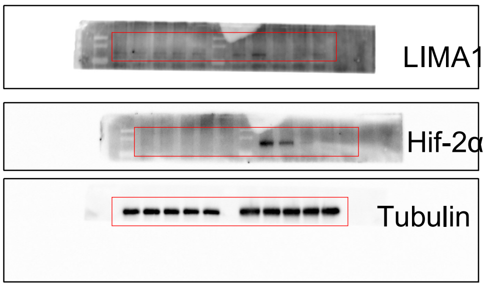

SNU449

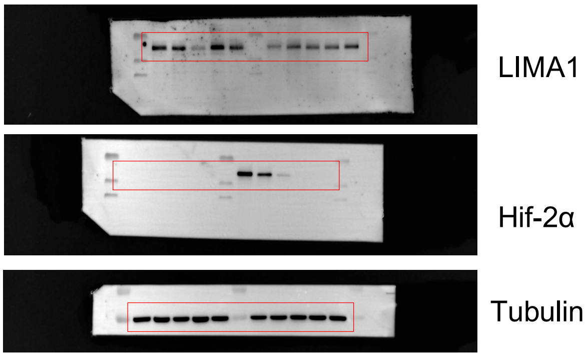

SMMC7721

Fig.S1 D

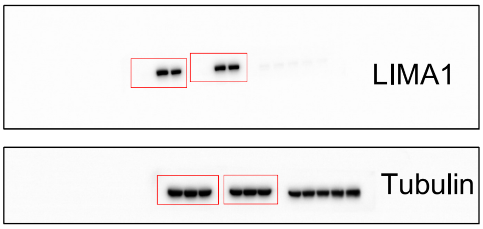

SMMC7721 SNU449

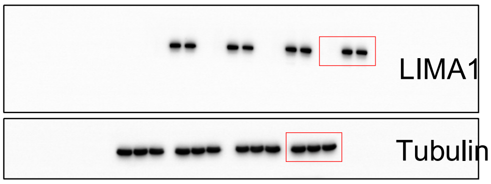

HUH7

Fig.S2 A

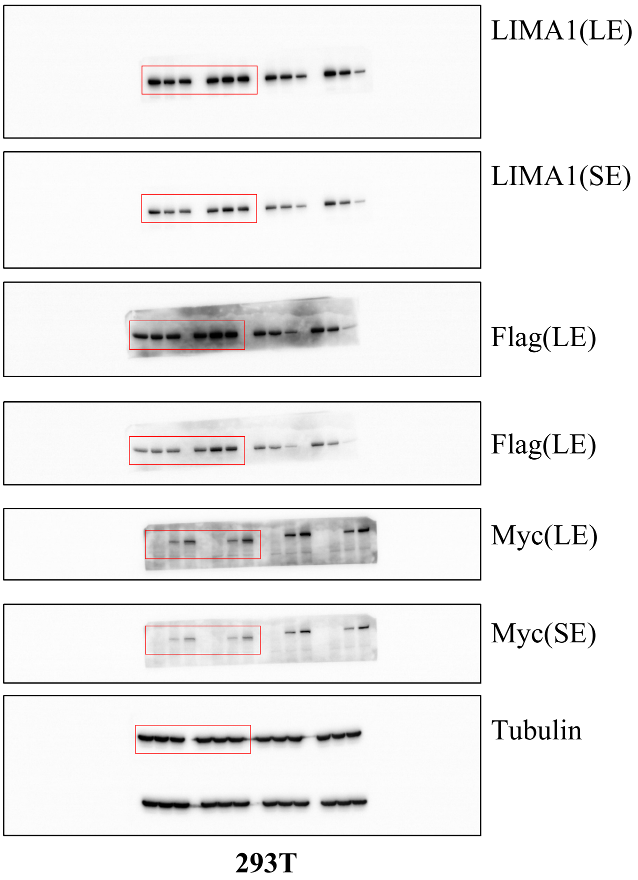

Fig.S3 A

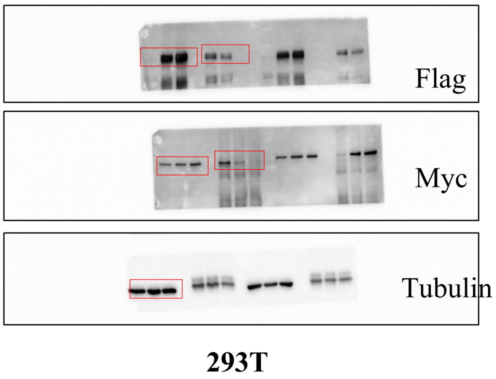

Fig.S3 C

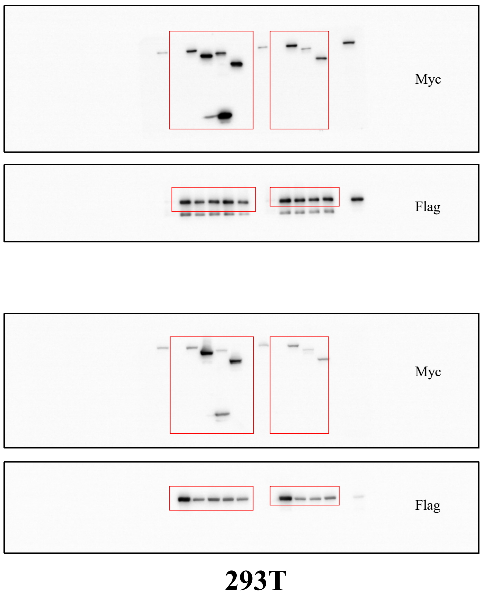

Supplement: Supplementary file 9 — Supplementary uncropped blots [file 41420_2024_2072_MOESM9_ESM.pdf]
